# DNA-binding by *Haemophilus influenzae *and *Escherichia coli *YbaB, members of a widely-distributed bacterial protein family

**DOI:** 10.1186/1471-2180-9-137

**Published:** 2009-07-13

**Authors:** Anne E Cooley, Sean P Riley, Keith Kral, M Clarke Miller, Edward DeMoll, Michael G Fried, Brian Stevenson

**Affiliations:** 1 Department of Microbiology, Immunology, and Molecular Genetics, University of Kentucky College of Medicine, Lexington, Kentucky, USA; 2 Paul Laurence Dunbar High School Math, Science and Technology Center, Lexington, Kentucky, USA; 3 Department of Chemistry, University of Kentucky, Lexington, KY, USA; 4 Department of Biology, University of Kentucky, Lexington, KY, USA; 5 Department of Molecular and Cellular Biochemistry, Center for Structural Biology, University of Kentucky College of Medicine, Lexington, Kentucky, USA; 6 Current address: Department of Surgery, Feinberg School of Medicine, Northwestern University, Chicago, Illinois, USA; 7 Current address: Dept of Microbiology, University of Chicago, Chicago, Illinois, USA; 8 Current address: Brown Cancer Center, University of Louisville, Louisville, Kentucky, USA

## Abstract

**Background:**

Genes orthologous to the *ybaB *loci of *Escherichia coli *and *Haemophilus influenzae *are widely distributed among eubacteria. Several years ago, the three-dimensional structures of the YbaB orthologs of both *E. coli *and *H. influenzae *were determined, revealing a novel "tweezer"-like structure. However, a function for YbaB had remained elusive, with an early study of the *H. influenzae *ortholog failing to detect DNA-binding activity. Our group recently determined that the *Borrelia burgdorferi *YbaB ortholog, EbfC, is a DNA-binding protein. To reconcile those results, we assessed the abilities of both the *H. influenzae *and *E. coli *YbaB proteins to bind DNA to which *B. burgdorferi *EbfC can bind.

**Results:**

Both the *H. influenzae *and the *E. coli *YbaB proteins bound to tested DNAs. DNA-binding was not well competed with poly-dI-dC, indicating some sequence preferences for those two proteins. Analyses of binding characteristics determined that both YbaB orthologs bind as homodimers. Different DNA sequence preferences were observed between *H. influenzae *YbaB, *E. coli *YbaB and *B. burgdorferi *EbfC, consistent with amino acid differences in the putative DNA-binding domains of these proteins.

**Conclusion:**

Three distinct members of the YbaB/EbfC bacterial protein family have now been demonstrated to bind DNA. Members of this protein family are encoded by a broad range of bacteria, including many pathogenic species, and results of our studies suggest that all such proteins have DNA-binding activities. The functions of YbaB/EbfC family members in each bacterial species are as-yet unknown, but given the ubiquity of these DNA-binding proteins among Eubacteria, further investigations are warranted.

## Background

**Figure 1:**
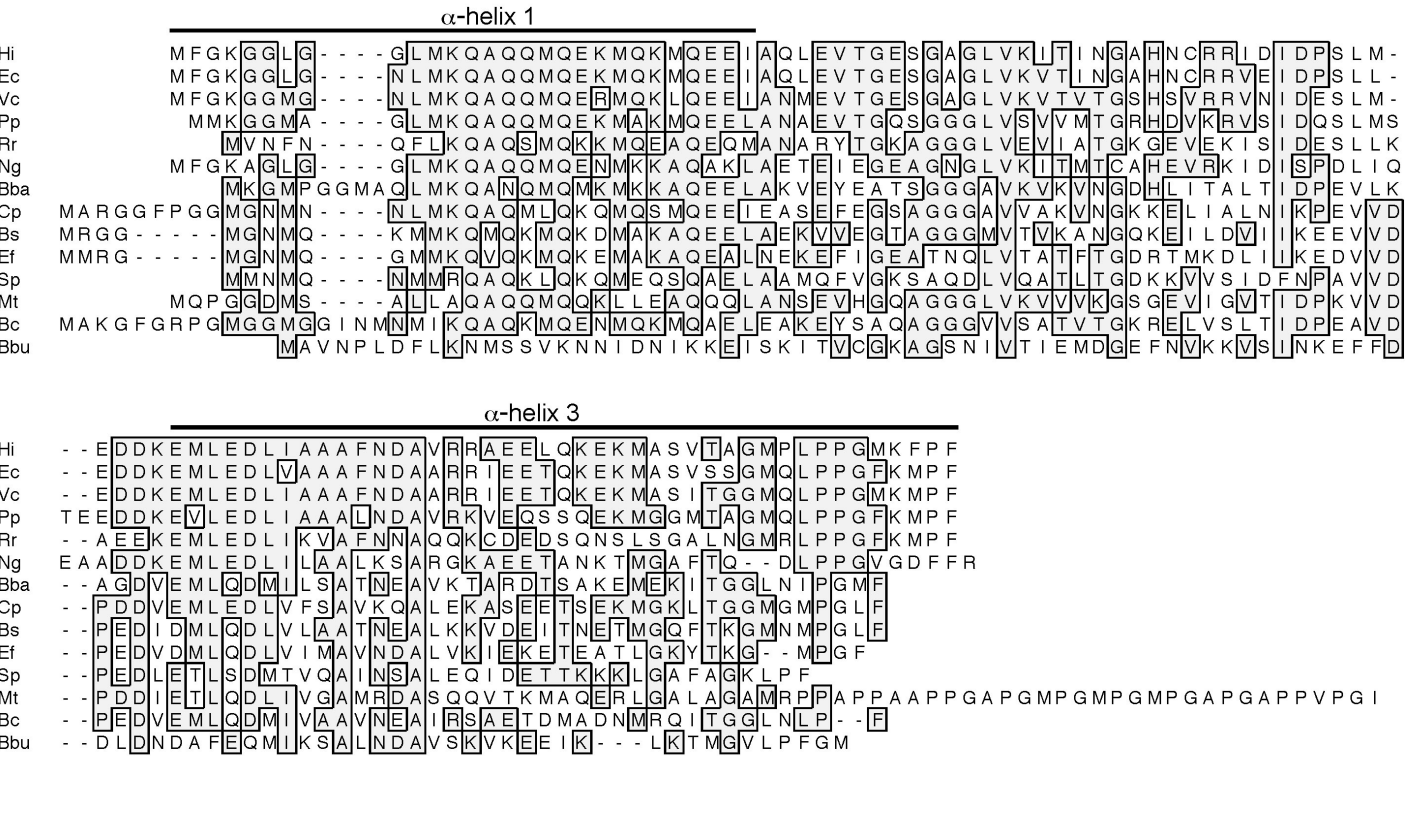
**Alignment of the predicted amino acid sequences of YbaB/EbfC orthologs of *H. influenzae *(Hi), *E. coli *(Ec), *Vibrio cholerae *(Vc), *Pseudomonas putida *(Pp), *Rickettsia rickettsiae *(Rr), *Neisseria gonorrhoeae *(Ng), *Bdellovibrio bacteriovorus *(Bba), *Clostridium perfringens *(Cp), *Bacillus subtilis *(Bs), *Enterococcus faecalis *(Ef), *Streptococcus pneumoniae *(Sp), *Mycobacterium tuberculosis *(Mt), *Bacteroides capillosus *(Bc), and *B. burgdorferi *(Bbu)**. Identical amino acids are boxed and shaded. Amino acid residues of YbaB_Ec _and YbaB_Hi _that comprise αlpha-helices 1 and 3 of their determined protein structures are identified.

Genome sequencing of diverse bacterial species has revealed widespread distribution of conserved gene products with as-yet unknown functions. Among these are a family of small proteins with approximate molecular masses of 12 kDa, which have been variously classed as "domain of unknown function" (DUF) 149, Pfam 2575 and COG-0718 [1]. Such genes have been identified in a wide variety of bacterial phyla, a list that includes many significant pathogens of humans, domestic animals and plants (Fig. 1).

After the genome sequence of *H. influenzae *strain KW20 rd (also known as *H. influenzae *Rd) was determined in 1995 [2], the "Structure 2 Function Project" was established to crystallize recombinant proteins from *H. influenzae *genes of unknown function http://s2f.umbi.umd.edu/. Among these orphan gene products was the *H. influenzae *DUF 149 group member annotated as open reading frame (ORF) HI0442, and tentatively named "YbaB" [3]. *H. influenzae *YbaB (YbaB_Hi_) crystallized as a homodimer, with the central portion forming 3 antiparallel β-strands, long α-helices at the amino- and carboxy-termini (α-helices 1 and 3, respectively), and a short α-helix bridging the β-folded region and α-helix 3 (α-helix 2). The two subunits of the homodimer interface at the β-strand region, α-helix 2 and the initial residues of α-helix 3, while α-helix 1 and the terminal portion of α-helix 3 project away from the dimerization region. This distinctive structure that has been described as resembling a set of tweezers [3]. Although the researchers who initially characterized YbaB_Hi _speculated that it may be a DNA-binding protein, studies conducted at that time failed to detect binding to any of their analyzed DNA probes [3].

The *Escherichia coli *chromosome carries an orthologous gene that has been referred to as "ORF 12" (Fig. 1) [4-6]. Recombinant *E. coli *YbaB (YbaB_Ec_) has also been crystallized and information about its unpublished three-dimensional structure is available on-line http://www.rcsb.org/pdb/explore.do?structureId=1PUG. The determined structures of YbaB_Ec _and YbaB_Hi _are nearly identical. A function for YbaB_Ec _appears not to have been investigated prior to the current work.

The spirochete *Borrelia burgdorferi *produces a protein named EbfC that shares 29% identical and 56% similar amino acids with YbaB_Hi _(Fig. 1). Our laboratories recently discovered that EbfC binds a specific DNA sequence 5' of the spirochete's *erp *loci [7-10]. Those results suggested that orthologous proteins may also be DNA-binding proteins. We therefore re-examined the properties of YbaB_Hi_, and found that it does bind to certain DNAs. YbaB_Ec _was also demonstrated to be a DNA-binding protein.

## Results and discussion

**Figure 2:**
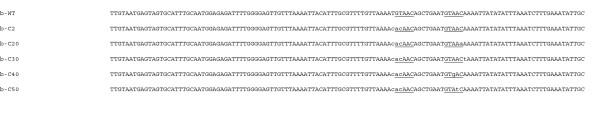
**Nucleotide sequences (5' to 3') of DNA probes used for EMSA in these studies, based on the operator 2 sequences of *B. burgdorferi erpAB ***[7,8,10]. Underlined nucleotides identify the wild-type (GTnAC) and mutated sequences to which *B. burgdorferi *EbfC will either bind or not bind, respectively (see Fig. 5). Mutated nucleotides are indicated by lower case letters. All probes used in EMSAs were labeled with a biotin moiety at the one 5' end.

**Figure 3:**
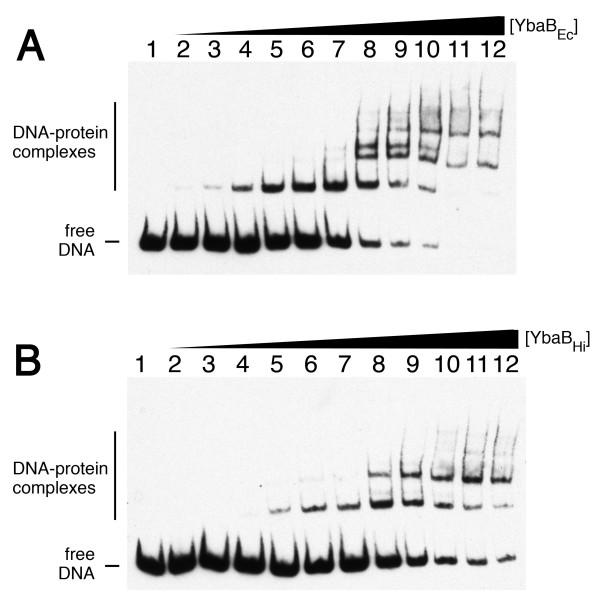
**YbaB_Ec _and YbaB_Hi _are DNA-binding proteins**. **(A) **Representative EMSA using labeled probe b-WT and increasing concentrations of recombinant YbaB_Ec_. Lane 1 lacked YbaB_Ec_, and lanes 2 through 12 contained 0.14, 0.21, 0.47, 0.93, 1.4, 1.8, 2.3, 4.7, 7.0, 9.4 or 12 μg/ml YbaB_Ec_, respectively. **(B) **Representative EMSA using labeled probe b-WT and increasing concentrations of recombinant YbaB_Hi_. Lane 1 lacked YbaB_Hi_, and lanes 2 through 12 contained 0.18, 0.26, 0.59, 1.2, 1.8, 2.3, 2.9, 5.9, 8.8, 12 or 15 μg/ml YbaB_Hi_, respectively.

The abilities of YbaB_Ec _and YbaB_Hi _to bind DNA were first tested using a labeled DNA probe corresponding to sequences surrounding *B. burgdorferi erpAB *Operator 2 (Fig. 2). This DNA was chosen because the *B. burgdorferi *YbaB ortholog, EbfC, binds specifically to sequences within that region of DNA [7,8]. Both the *E. coli *and *H. influenzae *orthologs bound this DNA probe, each forming multiple DNA-protein complexes (Fig. 3). The simplest interpretation of these data is that each ladder of gel bands represents a stoichiometric series with higher stoichiometry (lower mobility) products formed from lower stoichiometry (higher mobility) precursors as protein concentration is increased. Similar patterns have been reported for other molecular systems (e.g., lac repressor-DNA complexes and CAP-DNA complexes) for which this interpretation has been found to be correct [11,12]. The EMSA assay does not provide information about the nature of the macromolecular interactions that stabilize each protein-DNA complex. Thus while the formation of the first complex must involve protein-DNA contacts, the interactions that stabilize higher-order complexes may include protein-protein contacts or protein-DNA contacts or both. The simplest model, and the one we favor, is one in which similar mechanisms direct the binding of each protein unit to DNA or pre-existing protein-DNA complex. Affinity data for the first two binding steps (described below) are consistent with this picture, but do not rule out more heterogeneous binding mechanisms.

**Figure 4:**
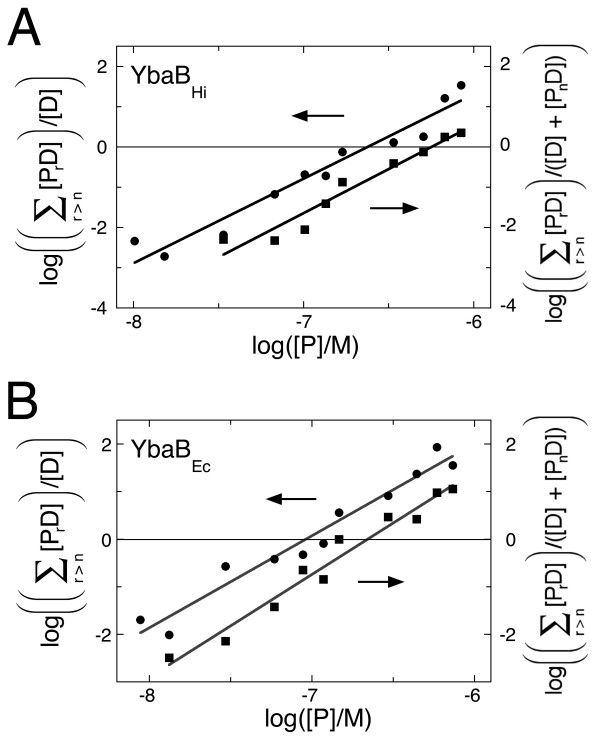
**Analysis of stoichiometries and affinities of YbaB_Ec _and YbaB_Hi _binding to b-WT DNA**. Data from the experiments shown in Fig. 3. **(A) **Binding of YbaB_Ec_. Symbols: (black circle), first binding step; (black square), second binding step. The lines are least-squares fits to Eqs 4 and 5, returning stoichiometry values of 1.93 ± 0.14 for the first binding step and 2.16 ± 0.14 for the second. From the logarithm of the free protein concentration at the midpoint of each binding transition we estimate that K_a,1 _= 1.7 ± 0.8 × 10^14 ^M^-2 ^and K_a,2 _= 2.9 ± 0.5 × 10^13 ^M^-2^. The ranges given for these parameters are 95% confidence limits calculated for the least squares fits. **(B) **Binding of YbaB_Hi_. Symbols: (black circle), first binding step; (black square), second binding step. The lines are least-squares fits to Eqs 4 and 5, returning stoichiometry values of 2.09 ± 0.16 for the first binding step and 2.18 ± 0.19 for the second. From the logarithm of the free protein concentration at the midpoint of each binding transition we estimate K_a,1 _= 1.7 ± 0.7 × 10^13 ^M^-2 ^and K_a,2 _= 3.0 ± 1.4 × 10^12 ^M^-2^. The ranges given for these parameters are 95% confidence limits calculated for the least squares fits.

Binding distributions were graphed (Fig. 4A) and analyzed according to Eqs. 3–5 (see the Methods section). These data are consistent with models in which 2 molecules of YbaB_Hi _bind free DNA to form the first complex, and in which the second binding step involves the concerted binding of 2 additional YbaB_Hi _molecules. For these binding models, the association constants for the first and second binding steps are K_a,1 _= 1.7 ± 0.7 × 10^13 ^M^-2 ^and K_a,2 _= 3.0 ± 1.4 × 10^12 ^M^-2^. Assuming equipartition of binding free energies, these values correspond to apparent, monomer-equivalent dissociation constants K_d,1 _= 2.4 ± 0.4 × 10^-7 ^M and K_d,2 _= 5.8 ± 1.0 × 10^-7 ^M. These values indicate that the two best YbaB_HI _binding sites on this DNA are of nearly equal affinity; the ~2-fold difference in affinity between first and second binding steps is just what would be expected on a statistical basis for independent binding to identical sites [13]. Parallel measurements were made for the binding of YbaB_Ec _to the b-WT DNA fragment (Fig. 4B). These data also indicate that 2 molecules of YbaB_Ec _bound free DNA to form the first complex and two more bound to form the second complex. The association constants for the first and second binding steps are K_a,1 _= 1.7 ± 0.8 × 10^14 ^M^-2 ^and K_a,2 _= 2.9 ± 0.5 × 10^13 ^M^-2^. Assuming equipartition of binding free energies as before, these correspond to monomer-equivalent dissociation constants K_d,1 _= 7.7 ± 0.4 × 10^-8 ^M and K_d,2 _= 1.9 ± 0.3 × 10^-7 ^M. As with the *H. influenzae *protein, the ~2-fold difference in affinity is what would be expected for independent binding to two identical sites. We note that these binding constants reflect binding under our standard *in vitro *conditions and should not be interpreted to represent the corresponding affinities for binding *in vivo*. None of our binding data suggests that either protein can bind DNA as a monomer. YbaB_Hi _and YbaB_Ec _proteins crystallized as dimers, and both previous sedimentation analyses and our gel filtration analyses indicated that YbaB_Hi _exists primarily as a homodimer in solution [data not shown and [3]]. Taken together, these data indicate that the homodimer is the basic unit of DNA-binding activity for this family of proteins.

In control experiments, purified YbaB proteins were treated either by incubation with 1 mg/ml proteinase K for 30 min or by heating in a boiling water bath for 10 min. EMSA of either protease-treated or boiled YbaB preparations did not yield reduced-mobility complexes or reduce the levels of free DNA probe (data not shown), demonstrating that the DNA-binding activity in the purified YbaB preparations was due to the native forms of the proteins.

**Figure 5:**
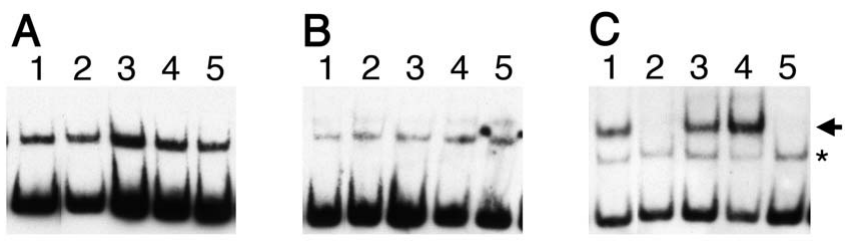
**Neither YbaB_Ec _nor YbaB_Hi _specifically binds the same nucleotide sequence as does *B. burgdorferi *EbfC**. For all panels, lanes 1 contain probe b-C2, lanes 2 contain probe b-C20, lanes 3 contain b-C30, lanes 4 contain b-C40, and lanes 5 contain b-C50. **(A) **YbaB_Ec_. **(B) **YbaB_Hi_. **(C) **EbfC, with the arrowhead indicating the specific EbfC-DNA complex and the asterisk indicating a non-specific EbfC-DNA complex [8,10].

*B. burgdorferi *EbfC binds specifically to the tetrad GTnAC, and mutation of any of those 4 bases eliminates specific DNA binding (Fig. 5, [8,10]). To assess the requirements for those nucleotides on YbaB_Ec _and YbaB_Hi _binding, EMSAs were performed using as probes either a derivative of *B. burgdorferi erpAB *operator 2 that contains only 1 consensus EbfC-binding site (probe b-C2) or that DNA containing single bp mutations (probes b-C20, 30, 40 and 50, Fig. 2). For each protein, a concentration of one half its K_d _was utilized in order to show either increases or decreases in binding. Note that both YbaB_Ec _and YbaB_Hi _produced one protein-DNA complex at these protein concentrations, whereas EbfC yielded two mobility complexes. Other studies from our laboratories demonstrated that the upper (more slowly migrating) EbfC-DNA complex represents specific binding to the GTnAC sequence, while the lower (more rapidly-migrating) complex reflects a sequence-nonspecific interaction [10]. None of the single mutations had any detectable effect on binding by either YbaB_Ec _or YbaB_Hi _(Fig. 5A &5B). Point mutations that disrupted the GTnAC sequence eliminated specific binding of EbfC, but did not affect non-specific binding by that protein (Fig. 5C).

**Figure 6:**
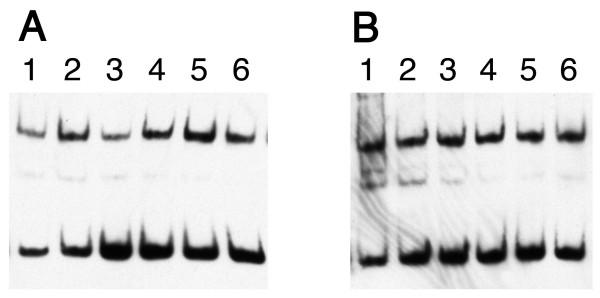
**Addition of increasing concentrations of poly(dI-dC) did not detectably alter DNA-binding by either YbaB ortholog**. **(A) **YbaB_Ec_. **(B) **YbaB_Hi_. For both panels, lanes 1 did not contain any poly(dI-dC), and lanes 2 through 6 contained 0.1, 0.5, 1, 2 or 4 ng per reaction, respectively.

The specificity of YbaB binding was further addressed by EMSA using progressively greater concentrations of poly(dI-dC), which acts as a competitor for non-specific DNA binding activities [14]. Addition of even 500-fold excesses of poly(dI-dC) had no measurable effect on either YbaB_Ec _or YbaB_Hi _binding to the *B. burgdorferi erpAB *operator 2 probe (Fig. 6).

A previous study did not detect binding of YbaB_Hi _to any tested DNA, leading to the conclusion that this protein does not bind DNA in a completely sequence-independent manner [3]. The present work demonstrated that YbaB_Hi_, and the homologous protein of *E. coli*, do bind to certain DNAs. EbfC, the orthologous protein of the spirochete *B. burgdorferi*, binds specifically to the DNA sequence GTnAC and, with a lower affinity, to DNA lacking that sequence [8,10]. The *E. coli *and *H. influenzae *YbaB proteins both exhibited preferences for certain tested DNA sequences, but neither showed the same high affinity for GTnAC as did the spirochetal ortholog. Both YbaB proteins also showed a marked preference for DNA derived from the *B. burgdorferi erpAB *promoter over poly(dI-dC). Such large differences in affinities for target and non-target sequences may account for the previous failure to detect DNA-binding by YbaB_Hi _[3]. These results suggest that YbaB_Ec _and YbaB_Hi _have higher affinities for some DNA sequences than for others, but whether those preferences depend upon a specific nucleotide sequence(s), A+T content, and/or DNA topology remain to be determined. The three-dimensional structure of dimeric YbaB resembles "tweezers", with α-helices 1 and 3 of each monomeric subunit protruding from the dimerization domains [3]. The spacing between the α-helical protrusions is approximately 15 Å at the base of the dimerization domain and approximately 22 Å at the distal ends of the α-helices [3], similar to the diameter of B-form duplex DNA (~20Å [3]). Site-directed mutagenesis studies of the orthologous *B. burgdorferi *EbfC demonstrated that certain amino acid substitutions in either α-helix 1 or 3 of EbfC eliminate DNA-binding, without affecting dimerization [10]. It is noteworthy that many of the α-helix 1 and 3 residues of EbfC are distinct from residues in both YbaB_Ec _and YbaB_Hi _(Fig. 1), consistent with the differences in DNA preferences between the *E. coli *and *H. influenzae *YbaB proteins and their spirochetal ortholog. YbaB/EbfC orthologs of other bacterial species likewise exhibit sequence variations in their α-helices 1 and 3, suggesting that they may also possess unique DNA-binding properties.

The function(s) of YbaB/EbfC proteins remains to be determined. Many bacterial *ybaB/ebfC *orthologs are located between *dnaX *and *recR*, a synteny that has led to suggestions of roles in DNA replication or recombination [3,5,6,15-18]. While the abilities of the examined orthologs to bind DNA may support those hypotheses, several lines of evidence suggest that YbaB/EbfC proteins perform functions that are independent of DNA recombination or replication. Proteomic analyses of cultured *H. influenzae *detected production of YbaB without accompanying production of DNA repair proteins [19]. A *ybaB recR *double mutant of *Streptomyces coelicolor *exhibited recombination defects that could be complemented with *recR *alone [18]. The *ybaB/ebfC *orthologs of some bacterial species are not linked to *recR *or any other recombination-related gene and some, such as the *B. burgdorferi*, do not even encode RecR [8,20]. Several bacteria, such as *H. influenzae*, have *ybaB *genes located distantly from their *dnaX *[2]. Moreover, some *ybaB *family genes can be transcribed independently of their upstream genes, using promoter elements within the 5' gene [4,6,21-23].

## Conclusion

We demonstrated that YbaB_Hi _is in fact a DNA-binding protein. It exhibits an element of specificity, in that the protein preferentially bound to *B. burgdorferi erp *Operator 2 DNA over poly-dI-dC and, apparently, the DNA sequences examined by an earlier research group [3]. Consistent with those data, the *E. coli *YbaB ortholog was also determined to be a DNA-binding protein. For both orthologs, the basic unit of DNA-binding is a homodimer, consistent with results from analyses of soluble proteins and crystallization data. The solved structures of YbaB_Ec _and YbaB_Hi _are distinct from any other known DNA-binding protein. Genes encoding orthologs of YbaB/EbfC proteins are found throughout the Eubacteria, including many important human pathogens, suggesting that these proteins perform important function(s). Thus, continued study of these unique proteins may provide insight regarding critical bacterial processes that might be exploited for infection control.

## Methods

### Bacterial gene sequences

Bacterial protein sequences orthologous to YbaB_Hi_, YbaB_Ec _and *B. burgdorferi *EbfC were identified by BlastP, using the predicted sequences of those three proteins as queries http://blast.ncbi.nlm.nih.gov/Blast.cgi. Amino acid sequences were aligned using Clustal X, with default parameters [24]. Orthologs from the following bacteria were chosen as representative of different bacterial classifications: the α proteobacterium *Rickettsia rickettsiae *(accession number NC_009882), the β proteobacterium *Neisseria gonorrhoeae *(NC_002946.2), the gamma proteobacteria *Vibrio cholerae *(NC_002505.1) and *Pseudomonas putida *(NC_010501.1), the delta proteobacterium *Bdellovibrio bacteriovorus *(NC_005363.1), the firmicutes *Clostridium perfringens *(NC_003366.1), *Bacillus subtilis *(NC_000964.2), *Enterococcus faecalis *(NC_004668.1), and *Streptococcus pneumoniae *(NC_003098.1), the actinomycete *Mycobacterium tuberculosis *(NC_000962.2), and the bacteroidete *Bacteroides capillosus *(NZ_AAXG02000011.1).

### Recombinant proteins

Recombinant YbaB_Hi _protein was produced from pET15b-HI0442 (a gift of Osnat Herzberg, University of Maryland) [3]. Recombinant YbaB_Ec _was produced by first PCR amplifying the *ybaB*_Ec _gene from total genomic DNA using the oligonucleotide primers 5'-CACCCGTGATTGAGGAGGAAACCTATG-3' and 5'-CAGCGGGCTGGTTTGCATCAG-3'. The resulting amplicon was cloned into pET200-TOPO (Invitrogen, Carlsbad, CA), and the insert completely sequenced on both strands. Recombinant *B. burgdorferi *EbfC was produced using the previously-described plasmid construct p462-M5 [8].

Each plasmid was individually used to transform *E. coli *Rosetta pLysS (Novagen, San Diego, CA), and production of recombinant proteins induced by addition of isopropylthiogalactopyranoside. Bacteria were lysed by sonication in 30 mM imidazole, 0.5 M NaCl, 20 mM NaPO_4_, pH = 7.4, and cleared by centrifugation. The recombinant proteins were purified using His-Trap HP columns and an AKTA-FPLC equipped with a UPC-900 UV absorbance monitor and a Frac920 fraction collector (GE Healthcare, Piscataway, NJ). Proteins were eluted with a constantly increasing gradient between the lysis buffer and 0.75 M imidazole, 20 mM NaPO4, 0.5 M NaCl, pH = 7.4. Proteins were then dialyzed against 1 × e0 buffer (50 mM Tris [pH = 7.5], 1 mM dithiothreitol, 1 mM phenylmethanesulfonyl fluoride, and 100 μl/l Tween-20). Glycerol was added to a final concentration of 10% (vol/vol), and aliquots were snap frozen in liquid nitrogen and stored at -80°C. Purity of protein preparations was assessed by sodium dodecylsulfate-polyacrylamide gel electrophoresis (SDS-PAGE), followed by staining with Coomassie brilliant blue. BCA (bicinchoninic acid) protein assays (Pierce, Rockford, IL), calibrated with bovine serum albumin (Pierce), were used to determine protein concentrations.

### Electrophoretic mobility shift assays (EMSA)

All EMSAs were performed at least three times. Biotin-labeled DNA probes were produced based upon the sequence of the *B. burgdorferi *strain B31 *erpAB *5'-noncoding DNA, to which the orthologous EbfC protein is known to bind [7,8,10]. Probe b-WT corresponds with bp -160 through -36 (relative to the start of translation) of the *erpAB *operon, and contains two consensus EbfC-binding sites [8,10] (Fig. 2). Probe b-WT was produced by PCR using oligonucleotide primers bio-A14A (5'-biotin-TTGTAATGAGTAGTGCATTTG-3') and R8 (5'-GCAATATTTCAAAGATTTAAA-3') from DNA template pBLS591 [7]. That same oligonucleotide primer pair was used to produce probe b-C2 from mutant template pSRJ-2, a derivative of pBLS591 in which EbfC-binding site II was changed to CACAACA (Fig. 2) [10]. Probes b-C20, b-C30, b-C40 and b-C50 were also produced using primers bio-A14A and R8, from mutant templates pSRJ-20, pSRJ30, pSRJ40 and pSRJ50, respectively, derivatives of pSRJ-2 in which single bp mutations were introduced to site I (Fig. 2) [10]. Each PCR reaction product was separated by agarose gel electrophoresis and DNA visualized by ethidium bromide staining. Amplicons were extracted from gels into nuclease-free water using Wizard SV (Promega, Madison, WI), and quantified by spectrophotometric determination of absorbance at 260 nm.

EMSAs were performed using 100 pM biotin-labeled DNA fragment and varying concentrations of purified recombinant YbaB_Ec _or YbaB_Hi_. Binding conditions consisted of 50 mM Tris-HCl (pH = 7.5), 1 mM dithiothreitol, 8 μl/ml protease inhibitor (Sigma-Aldrich, St. Louis, MO), 2 μl/ml phosphatase inhibitor cocktail II (Sigma-Aldrich), and 10% glycerol. Protein and DNA were mixed together, in final volumes of 10 ml, and allowed to proceed toward equilibrium for 20 minutes at room temperature, then subjected to electrophoresis through 6% DNA retardation gels (Invitrogen) for 9000 V-min. DNA was electrotransferred to Biodyne B nylon membranes (Pierce), cross-linked by ultraviolet light, and biotinylated DNA detected using Chemiluminescent Nucleic Acid Detection Modules (Pierce).

Competition for DNA binding by poly(dI-dC) was assessed using the above binding conditions, 2 fmol (0.082 ng) labeled probe b-WT and either 1.2 μg/ml YbaB_Ec _or 2.1 μg/ml YbaB_Hi_. After 20 min incubation at room temperature, either no or 0.1, 0.5, 1, 2 or 4 ng poly(dI-dC) was added to each tube, followed by an additional 20 min incubation at room temperature. DNA-protein mixtures were subjected to electrophoresis and detection as described above.

### Binding analyses

Exposed films were scanned in 8 bit depth at 1200 dpi resolution using Image J 1.37 v http://rsbweb.nih.gov/ij/. Band intensities were converted into mole fractions as previously described [11]. Binding was analyzed according to a model in which several molecules of protein can bind the target DNA according to the general mechanism(1)

here n, m and q are n numbers of protein monomers that associate at the first, second and third binding steps, characterized by association constants K_a,1_, K_a,2 _and K_a,3_, respectively. As indicated by the ellipsis, this model can include > 3 binding steps, as necessary. For the first binding step(2)

When not complicated by subsequent binding events, the evaluation K_a,1 _can be done according to standard procedures [12,25]. However, when higher-stoichiometry complexes accumulate before the first step reaches saturation, as is the case for the binding reactions shown in Fig. 3, it is necessary to account for all of the species in the equilibrium mixture that are formed from P_n_D. When this is done, the equilibrium constant for the first binding step becomes(3)

Here the subscript r denotes the protein stoichiometry of the corresponding complex. Rearranging Eq. 3 and taking logs gives(4)

Thus, a graph of  as a function of log [P] will have a slope equal to the stoichiometry n and an x-intercept at which -n log [P] = log K_a_. For the binding of m protein molecules to a P_n_D complex, the corresponding expression is(5)

It is important to note that in this approach, values of stoichiometry and equilibrium constant are not fully independent (fitted values of K_a _and n are related by -n log [P] = log K_a_). As a result, the parameters returned are the most likely values (in the least squares sense) that are internally-consistent. A similar analysis strategy has been described previously [12].

In studies of this kind, accurate measurement of K_a _values require good estimates of the free protein concentration, [P]. In the present experiments, the protein concentrations (range ~10^-8 ^M to ~10^-6 ^M) exceeded by far the total DNA concentration (10^-10 ^M). Thus, even in the presence of additional DNA binding (up to ~10 protein molecules/DNA), free protein concentration [P] is well-approximated by the total protein concentration, [P]_total_.

### Size-exclusion chromatography

A Superdex 75 10/300 GL column (GE Healthcare) was prepared with a mobile phase consisting of 200 mM NaCl, 50 mM Tris-HCl (pH 7.5), and 1% (vol/vol) glycerol. The column was run with a flow rate of 0.20 ml per min using a Waters 600 pump and controller equipped with a Waters 996 photodiode array UV/Vis detector (Waters, Milford, MA). A calibration curve was created using an MW-GF-70 low-molecular-weight calibration kit (Sigma-Aldrich, St. Louis, MO), and the void volume, *V*_0_, was determined by injection of 200 μl of 1 mg/ml blue dextran in elution buffer with 5% glycerol. The remaining protein standards, bovine lung aprotinin (6.5 kDa), horse heart cytochrome *c *(12.4 kDa), bovine carbonic anhydrase (29 kDa), and bovine serum albumin (66 kDa), were individually prepared in elution buffer with 5% glycerol to total concentrations of 0.3 mg/ml each, and the volume with which the protein eluted, V_e_, was determined. The molecular-mass calibration curve was generated by plotting the log (molecular mass) versus V_e_/V_o _(5). A 200-μl sample of recombinant YbaB_Hi _(approximately 0.2 mg/ml) was then injected and its elution profile compared to the established curve to determine molecular masses of each elution peak.

## Authors' contributions

AEC, ED, MGF and BS designed the experiments. AEC, SPR and KK performed EMSA analyses. MCM and ED conducted size exclusion chromatography. AEC, SPR, ED, MGF and BS interpreted the results. All authors read and approved the manuscript.
